# Acute exposure to clozapine and sodium valproate impairs oxidative phosphorylation in human cardiac mitochondria

**DOI:** 10.1016/j.toxrep.2025.101990

**Published:** 2025-03-05

**Authors:** Amanda Groenewald, Kathryn E. Burns, Malcolm D. Tingle, Marie-Louise Ward, Amelia S. Power

**Affiliations:** a Department of Physiology, Faculty of Medical and Health Sciences, The University of Auckland, Auckland, New Zealand; b Department of Pharmacology and Clinical Pharmacology, Faculty of Medical and Health Sciences, The University of Auckland, Auckland, New Zealand

**Keywords:** Schizophrenia, Clozapine, Sodium valproate, Cardiotoxicity, Myocardial bioenergetics, Mitochondria, Oxidative phosphorylation

## Abstract

The only antipsychotic that is approved and recommended for the treatment of otherwise treatment-resistant schizophrenia is clozapine (CLZ). Unfortunately, CLZ can cause serious cardiotoxicities such as myocarditis and cardiomyopathy. The co-administration of sodium valproate (VPA) during initiation is a well-established risk factor for the development of CLZ-induced myocarditis. However, the mechanisms behind these cardiac adverse effects and the role of VPA co-administration are not understood. Preliminary evidence for the development of cardiac mitochondrial dysfunction has previously been reported in both rodent models and immortalised cell lines. This investigation aimed to determine the functional effects of CLZ and VPA on human cardiac mitochondria to improve the current understanding of how cardiotoxicity develops. Small samples of human atrial tissue from consenting patients undergoing a coronary artery bypass grafting procedure were freshly collected and utilised to investigate the acute effects of each drug on mitochondrial O_2_ consumption using high-resolution respirometry. Both drugs significantly decreased mitochondrial O_2_ consumption by a magnitude of 32 % following CLZ exposure, 25 % following VPA exposure, and 25 % following combined CLZ+VPA exposure during complex I- and II-linked oxidative phosphorylation. These results demonstrate acute bioenergetic dysfunction with exposure to both drugs, alone and in combination. We propose that cardiac mitochondria become a key focus in future research seeking to improve the risk-predictive, diagnostic, and treatment guidelines surrounding CLZ-induced cardiotoxicity.

## Highlights

• Clozapine and sodium valproate can cause acute dysfunction in human cardiac mitochondria.
• Acute exposure to clozapine impaired O_2_ consumption during oxidative phosphorylation (OXPHOS) through complexes I and II.
• Acute exposure to sodium valproate, with or without clozapine, impaired OXPHOS efficiency through complex I.

## 1 Introduction

Schizophrenia is a debilitating mental health disorder characterised by positive symptoms (such as hallucinations and delusions), negative symptoms (such as apathy and avolition), as well as progressive cognitive decline in the absence of effective treatment [1]. Approximately one in three patients with schizophrenia are considered “treatment-resistant”, which is defined by the inadequate management of symptoms following the trial of at least two antipsychotic drugs [2]. Clozapine (CLZ) is currently the only drug recommended for treatment-resistant patients [3]. It is effective for approximately 30 % of these patients and, despite being given to those with more severe illness, has been associated with better outcomes concerning hospitalisation, treatment discontinuation, and symptom severity [4], [5], [6]. CLZ is also one of the only antipsychotic drugs shown to significantly decrease suicidality [7], [8].

Despite its efficacy, initiation of CLZ treatment is typically delayed, which has been associated with an impaired response to treatment and a greater economic burden [9], [10], [11]. The reluctance to prescribe CLZ can be attributed to its many adverse effects including agranulocytosis, seizures, gastrointestinal hypomotility, diabetes, and cardiotoxicity [12], [13]. Most of these can be managed using adjunct drug therapies or, in the case of agranulocytosis, mandatory blood count monitoring [14], [15]. However, there is currently a lack of clear guidelines for the detection and treatment of CLZ-induced cardiotoxicity [16]. It is typically diagnosed either using non-specific indicators of inflammation and cardiomyocyte injury such as elevated plasma troponin and TNF-α levels, or by echocardiogram. Both approaches rely on a clinician suspecting cardiotoxicity when patients present with nonspecific symptoms [17], [18]. This means that patients are unlikely to be diagnosed and treated quickly. Beyond cessation of CLZ, the treatment of cardiotoxicity would typically involve the empirical use of beta-blockers, corticosteroids, and/or diuretics [19].

The major cardiotoxic effects of CLZ include myocarditis, which is usually reported within two to eight weeks of treatment initiation, and cardiomyopathy, which can take anywhere between six and nine months to present [19], [20]. CLZ treatment has been reported to be associated with a 0.7 % and 0.6 % incidence of myocarditis and cardiomyopathy, respectively, in a meta-analysis involving patients across the globe [21]. Mortality rates as high as 64 % for CLZ-induced myocarditis and 24 % for CLZ-induced cardiomyopathy have previously been reported [22]. The only established risk factors for the development of CLZ-induced cardiotoxicity are the faster titration onto the drug and the concomitant administration of sodium valproate (VPA) [23], [24]. VPA is often given alongside CLZ for its mood stabilising and anticonvulsant effects [25], [26], [27]. The lack of clear, evidence-based treatment guidelines for CLZ-induced cardiotoxicity may result from its underlying mechanisms being unknown, challenging the development of targeted treatment strategies. The role of VPA in increasing the risk of developing CLZ-induced cardiotoxicity is also unknown. A clearer understanding of these processes will therefore be essential in improving treatment, diagnostic, and risk-predictive guidelines surrounding the safe use of these drugs.

Recently, evidence has emerged that CLZ may directly impair mitochondrial function. For instance, acute exposure of several cell lines and isolated mitochondria to high concentrations of CLZ has been shown to depolarise the mitochondrial membrane and impair O_2_ consumption [28], [29], [30], [31]. VPA is known to impair mitochondrial function, inhibiting oxidative phosphorylation (OXPHOS) and fatty acid oxidation in rodent hepatocytes and isolated hepatic mitochondria [32], [33], [34], [35], [36]. However, VPA alone is not considered to be cardiotoxic, and some studies suggest it could be cardioprotective prior to an ischaemic event [37]. For that reason, VPA’s inhibitory effect on fatty acid oxidation is thought to be specific to hepatic mitochondria [38]. However, it may have a subclinical effect on cardiac mitochondria that exacerbates the adverse effects of CLZ.

The heart requires a continuous supply of ATP to fuel excitation-contraction coupling and relaxation of cardiomyocytes during normal heart function. Mitochondria occupy 35–40 % of the cardiomyocyte volume, supplying up to 95 % of the heart’s ATP requirements [39]. Impaired ATP synthesis following CLZ exposure would impact cardiac systolic and diastolic function and may lead to the development of cardiomyopathy [40]. Mitochondria also have a role in the development of necrotic and apoptotic cell death [41]. If CLZ exposure causes severe mitochondrial dysfunction, it could induce the release of inflammatory mediators that precede the development of myocarditis. However, the mitochondrial effects of CLZ and VPA have not been investigated in the human heart. This study therefore aimed to determine the acute effects of CLZ and VPA exposure, alone and in combination, on the mitochondrial function of freshly excised human cardiac tissue.

## 2 Methods

Unless otherwise specified, all chemicals were purchased from Sigma-Aldrich Co. (Merck KGaA, Darmstadt, Germany).

### 2.1 Ethics approval

The collection of human right atrial appendage tissue for this study was approved by the Health and Disability Ethics Committee of New Zealand (2023 PR 6432) and the Auckland District Health Board Research Review Committee (A+7593). All patient data collected for this investigation was de-identified.

### 2.2 Human sample collection

Tissue samples were collected from consenting patients undergoing a routine coronary artery bypass grafting procedure at Auckland City Hospital, New Zealand. During surgery, the patient’s right atrial appendage was cannulated, and a small piece of tissue was removed from the border of the incision (∼ 0.5 × 2 cm). The sample was immediately placed in 100 mL Krebs-Henseleit buffer (118 mM NaCl, 4.75 mM KCl, 1.18 mM MgSO_4_.7H_2_O, 1.18 mM KH_2_PO_4_, 24.8 mM NaHCO_3_, and 11 mM D-glucose) containing 20 mM butanedione monoxime and 0.25 mM CaCl_2_, with pH maintained at 7.4 by bubbling with carbogen (95 % O_2_, 5 % CO_2_) during transport back to the laboratory.

### 2.3 Preparation of permeabilised heart fibres

Once in the laboratory, two to three tissue blocks of approximately 10 mg were taken from each sample and stored on ice in biopsy preservation solution (BIOPS: 2.77 mM CaK_2_EGTA, 7.23 K_2_EGTA, 5.77 mM Na_2_ATP, 6.56 mM MgCl_2_.6H_2_O, 20 mM taurine, 15 mM Na_2_phosphocreatine, 0.5 mM dithiothreitol, and 50 mM MES hydrate) to be used in a time control (CTRL) and at least one acute drug exposure assay [42], [43]. Tissue blocks were teased apart into fibre bundles (∼ 300 µg) to enhance the diffusion of substrates and O_2_ throughout. Care was taken to exclude connective and fatty tissue from the fibre bundles.

The fibres were then transferred to 1 mL of fresh BIOPS and plasma membranes were permeabilised by the addition of saponin (5 mg.mL^−1^) before agitation on a rocker table for 30 minutes at 4 °C [44], [45]. Fibres were then transferred to 1 mL of mitochondrial respiration medium (MiR05: 0.5 mM EGTA, 3 mM MgCl_2_.6H_2_O, 60 mM K-lactobionate, 20 mM taurine, 10 mM KH_2_PO_4_, 20 mM HEPES, 110 mM sucrose, and 1 g.L^−1^ essential fatty acid-free bovine serum albumin) and agitated for 10 minutes to wash out the saponin and disperse the cytosolic contents. This washout step was repeated twice before the fibres (∼ 3–5 mg) were added to each 2 mL chamber of an oxygraph-O2K high-resolution respirometer (Oroboros Instruments, Austria).

### 2.4 High-resolution respirometry

Measures of O_2_ consumption normalised to tissue wet weight (pmol.s^−1^.mg^−1^) were obtained using DatLab 7 software (Oroboros Instruments, Innsbruck, Austria). Either MiR05 (CTRL), CLZ (15 µM), VPA (6 mM), or a combination (CLZ+VPA) were then added to the MiR05-filled chambers containing the tissue, followed by a 30-minute drug incubation before the start of each assay. CLZ (> 98 %) was prepared in methanol (> 99 %) to make a stock solution of 2 mM and VPA (> 97.5 %) in MiR05 to make a stock solution of 120 mM. Experiments were conducted (n = 4) comparing the effects of the blank CTRL utilised in this experimental series to the CLZ vehicle, methanol, to ensure that there was no difference between these in the respiratory measures made (see Fig. S1 for details).

**Fig. 1:**
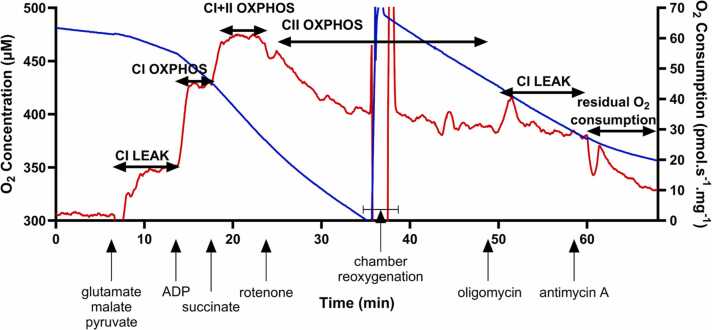
Representative respirometry trace. Representative trace showing the simultaneous measurement of O_2_ concentration (blue, µM) and O_2_ consumption (red, pmol.s^−1^.mg^−1^) from permeabilised human right atrial appendage fibres. Vertical arrows indicate the addition of substrates/inhibitors and horizontal arrows indicate the resulting respiratory pathway/state induced.

Following the drug incubation period, a series of substrates and inhibitors were sequentially titrated into each of the oxygraph chambers to induce both phosphorylating (OXPHOS) and non-phosphorylating (LEAK) respiration through complex I (CI) and/or complex II (CII) of the electron transport system, in an assay modified from [45], [46]. This included: 10 mM glutamate, 2 mM malate, and 2 mM pyruvate (CI-linked LEAK respiration); followed by 5 mM Mg^2+^-ADP (CI-linked OXPHOS); 10 mM succinate (CI- and CII-linked OXPHOS); 10 µM rotenone (CII-linked OXPHOS); 5 µM oligomycin (CII-linked LEAK respiration); and 5 µM antimycin A (residual O_2_ consumption). Measures of O_2_ consumption were allowed to reach a steady-state plateau following the titration of each substrate and inhibitor, which typically took between two and 10 minutes (Fig. 1).

### 2.5 Data analysis

A stable section of the signal following the addition of antimycin A was subtracted from each of the respiratory states measured to correct for residual O_2_ consumption. Each tissue sample collected supplied enough permeabilised fibres for two to three assays, including one CTRL and at least one of the drug conditions. Each assay was conducted in duplicate [45]. O_2_ consumption measures were extracted from DatLab 7 (Oroboros Instruments, Innsbruck, Austria) and averaged across the two chambers for each respiratory state and pathway. Sample sizes were 10–11 for each drug exposure group, varying when the amount of tissue available was limited. NET OXPHOS efficiency was estimated as the measured O_2_ consumption during OXPHOS corrected for LEAK respiration through a particular pathway, relative to the total measured O_2_ consumption during OXPHOS through the same pathway, as per the following equation [46]:

(1) $$\mathrm{NET OXPHOS efficiency}=1-\frac{\left(\mathrm{OXPHOS}-\mathrm{LEAK}\right)}{\mathrm{OXPHOS}}$$

Statistical analysis was undertaken in GraphPad Prism 10 (GraphPad Software, Boston, USA) using a repeated measures two-way ANOVA with Holm-Šídák’s multiple comparisons testing to make comparisons between drug exposures and their paired CTRL within each respiratory state. Patient characteristics between each group were compared using a one-way ANOVA with Tukey’s multiple comparisons test for continuous variables, and a Chi-square test for categorical variables. Statistical significance was defined where the likelihood of type 1 error was equal to or less than 5 % (P ≤ 0.05). All measures were reported as mean ± standard deviation unless otherwise specified.

## 3 Results

### 3.1 Patient characteristics

A total of 24 patient samples were utilised in this study. Samples came from patients that were on average 67 ± 9 years old, with a BMI of 27.90 ± 4.84 kg.m^−2^. Most of these patients were male (20/24 samples) and of European ethnicity (15/24 samples). Almost half of the samples (10/24) came from patients who had been diagnosed with Type 2 Diabetes. None of the patients had been prescribed either CLZ or VPA. Each experimental group utilised right atrial appendage samples from 10 to 11 patients. Patient characteristics and comorbidities were equally distributed between groups, and measures of O_2_ consumption during combined CI- and CII-linked OXPHOS were not different between time controls from each group (Table 1).

**Table 1:** Patient characteristics and comorbidities. Patient characteristics and medications from human right atrial appendage samples utilised. Data shown are reported as mean ± SD, or as a fraction of the total number of samples. Each sample was utilised in a control (CTRL, n = 24) assay and at least one acute drug exposure assay, by which three groups were defined (CLZ, VPA, or CLZ+VPA). In this way, tissue from a single patient sample could contribute to multiple groups.

	CLZ (n = 11)	VPA (n = 10)	CLZ+VPA (n = 10)	P value
Age (years)	66 ± 9	68 ± 8	65 ± 11	0.60
Gender (male)	9/11	9/10	8/10	0.81
BMI (kg.m^−2^)	28.69 ± 4.22	28.26 ± 4.94	27.29 ± 5.83	0.78
Ethnicity (European)	8/11	7/10	5/10	0.50
Smoking status (Ex-)	5/11	5/10	4/10	0.90
Diabetic (Type 2)	4/11	2/10	6/10	0.18
Hypertensive	8/11	7/10	7/10	0.99
Hyperlipidaemic	6/11	6/10	6/10	0.96
Ejection Fraction (%)	46.55 ± 11.42	46.45 ± 15.38	37.36 ± 9.72	0.60
CTRL CI+II OXPHOS (pmol.s^−1^.mg^−1^)	51.94 ± 17.60	46.45 ± 15.38	37.36 ± 9.72	0.09
Medications:				
ACE inhibitors	4/11	3/10	6/10	0.36
AT1 blockers	2/11	3/10	1/10	0.52
Βeta-blockers	7/11	6/10	7/10	0.89
Ca^2+^channel blockers	4/11	4/10	3/10	0.89
Diuretics	3/11	2/10	1/10	0.61
Nitrates	5/11	3/10	3/10	0.69
Lipid lowering drugs	7/11	7/10	10/10	0.11

### 3.2 Respirometry

**Fig. 2:**
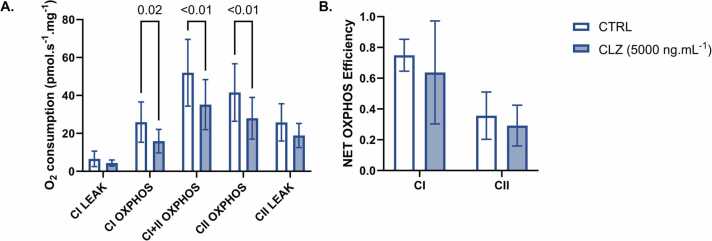
Mitochondrial respiration in permeabilised human atrial fibres following acute CLZ exposure. A. Following 30 min of exposure to CTRL (clear bars) or CLZ (filled bars), CI- and/or CII-linked LEAK or OXPHOS were induced by the sequential titration of substrates and inhibitors before measuring O_2_ consumption. Residual O_2_ consumption following the addition of antimycin A was subtracted from each measure. B. NET OXPHOS efficiency through CI and CII was estimated from these measures (see Eq. 1). Data are presented as mean ± SD (n = 11). Significant differences (P ≤ 0.05) are reported from a repeated measures two-way ANOVA with Holm-Šídák’s multiple comparisons tests.

Acute exposure of atrial fibres to CLZ did not affect LEAK respiration. However, CLZ decreased respiration by 39 %, 32 %, and 33 % during CI-, combined CI+II, and CII-linked OXPHOS, respectively, compared to CTRL (Fig. 2A). No significant change was observed in the estimated CI- or CII-linked NET OXPHOS efficiency (Fig. 2B).

**Fig. 3:**
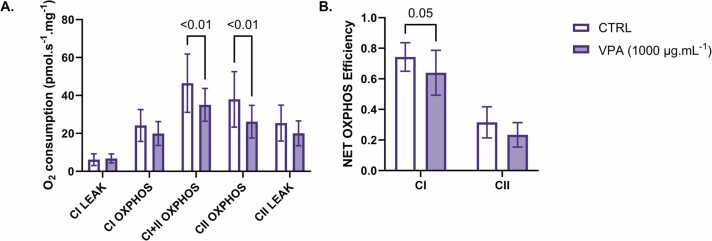
Mitochondrial respiration in permeabilised human atrial fibres following acute VPA exposure. A. Following 30 min of exposure to CTRL (clear bars) or VPA (filled bars), CI- and/or CII-linked LEAK or OXPHOS were induced by the sequential titration of substrates and inhibitors before measuring O_2_ consumption. Residual O_2_ consumption following the addition of antimycin A was subtracted from each measure. B. NET OXPHOS efficiency through CI and CII was estimated from these measures (see Eq. (1)). Data are presented as mean ± SD (n = 10). Significant differences (P ≤ 0.05) are reported from a repeated measures two-way ANOVA with Holm-Šídák’s multiple comparisons tests.

Exposure to VPA caused O_2_ consumption to decrease by 25 % and 31 % during combined CI+II-, and CII-linked OXPHOS, respectively, compared to CTRL (Fig. 3A). This was accompanied by a significant decrease in the estimated NET OXPHOS efficiency of CI-linked respiration in these fibres (Fig. 3B).

**Fig. 4:**
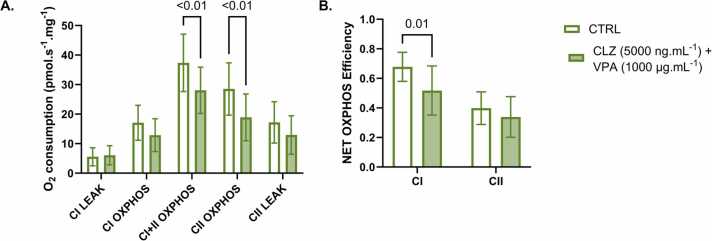
Mitochondrial respiration in permeabilised human atrial fibres following combined CLZ and VPA exposure. A. Following 30 min of exposure to CTRL (clear bars) or CLZ+VPA (filled bars), CI- and/or CII- linked LEAK or OXPHOS were induced by the sequential titration of substrates and inhibitors before measuring O_2_ consumption. Residual O_2_ consumption following the addition of antimycin A was subtracted from each measure. B. NET OXPHOS efficiency through CI and CII was estimated from these measures (see Eq. (1)). Data are presented as mean ± SD (n = 10). Significant differences (P ≤ 0.05) are reported from a repeated measures two-way ANOVA with Holm-Šídák’s multiple comparisons tests.

Similarly, exposure to CLZ and VPA together caused the mean O_2_ consumption to decrease by 25 % and 34 % during combined CI+II- and CII-linked OXPHOS, respectively, when compared to CTRL (Fig. 4A). This was also accompanied by a significant decrease in the estimated NET OXPHOS efficiency of CI-linked respiration in these fibres (Fig. 4B).

## 4 Discussion

This study is the first to demonstrate that acute CLZ exposure impairs OXPHOS in human cardiac mitochondria. CLZ decreased O_2_ consumption during OXPHOS through both CI- and CII-linked respiration (Fig. 2A). However, the drug did not significantly affect NET OXPHOS efficiency through either pathway (Fig. 2B). Previous studies of CLZ’s mitochondrial effects suggested that it would uncouple mitochondria, inducing LEAK respiration and inhibiting OXPHOS, thereby decreasing NET OXPHOS efficiency. For example, CLZ caused a collapse in the mitochondrial membrane potential of isolated rat ventricular cardiomyocytes and cultured mouse myoblasts [28], [29], [30]. Similarly, CLZ significantly decreased O_2_ consumption across all measured respiratory states in human lymphoblastoid cells [47]. CLZ partially inhibited CI-linked OXPHOS in isolated pig brain mitochondria and induced CII-linked OXPHOS at lower concentrations before inhibiting it at higher concentrations [48]. However, CLZ appeared to have no significant effect on LEAK respiration or OXPHOS efficiency in human cardiac mitochondria, implying that it impairs OXPHOS without necessarily affecting mitochondrial coupling.

These results may instead indicate a directly inhibitory effect of CLZ on CI and CII, or the upstream mitochondrial enzymes involved in the generation of NADH and FADH_2_ [49]. The inhibition of O_2_ consumption during OXPHOS only may also suggest that CLZ inhibits the ATP synthase itself [49]. Further investigation of the direct effects of CLZ on individual respiratory complexes could be made using specific enzyme assays and tailored high-resolution respirometry protocols [50], [51]. High-resolution respirometry assays could also be integrated with simultaneous measures of mitochondrial ATP production or membrane potential to provide an integrated measure of intact mitochondrial function [52].

Importantly, the previous studies mentioned utilised extremely high concentrations of CLZ. For instance, the inhibitory effect of CLZ on CII-linked OXPHOS in isolated pig brain mitochondria was not observed before concentrations approximately 30 times greater than the therapeutic alert level were achieved[48], [53]. The concentration of CLZ used in our investigation, while still supratherapeutic, was only five times greater than the accepted alert level, and less than half of the highest concentration to have been reported in overdose [53], [54]. It is also important to note that previous studies utilised isolated mitochondria and immortalised cell lines, rather than permeabilised fibres that provide a physiologically relevant model of intact mitochondria *in situ* [55]. The absence of mitochondrial uncoupling in our study may be in part due to the differences in species and mitochondrial preparations, but the higher concentrations used by others would likely have exacerbated any drug effects.

The heart is especially reliant on OXPHOS, possibly explaining why human atrial tissue may be more susceptible to CLZ’s mitochondrial effects than other cellular preparations, leading to dysfunction at lower concentrations of exposure [56], [57]. However, the samples utilised in this study may also have been predisposed to dysfunction on account of the comorbidities of the patient cohort, including ischemic heart disease and diabetes (Table 1)[58], [59], [60], [61]. Importantly, the characteristics of these patients were very similar to those previously reported in CLZ-taking patients, which highlights the importance of understanding CLZ’s role in exacerbating pre-existing cardiac dysfunction [62].

Our investigation also provides the first evidence of an acute effect of VPA alone on human cardiac mitochondria. VPA exposure decreased O_2_ consumption through combined CI and CII-, as well as CII-linked OXPHOS (Fig. 3A). This could be indicative of an inhibitory effect of VPA on CII or the upstream mitochondrial enzymes responsible for the production of FADH_2_ [49], [63]. While VPA had no significant effect on NET OXPHOS efficiency through CII, it was decreased through CI (Fig. 3B). These findings suggest that VPA may impair ADP phosphorylation efficiency since CI-linked respiration contributes more to the movement of protons across the inner mitochondrial membrane per molecule of O_2_ consumed [40]. VPA is metabolised through β-oxidation, which may cause it to induce O_2_ consumption via CI or the electron-transferring flavoprotein complex, which may mask its effects on CI-linked OXPHOS [46], [64], [65]. Further investigation of VPA’s effects on mitochondrial membrane potential and ATP production, as well as its specific effect on the oxidation of fatty acids, would be necessary to confirm this.

Our observation of acute dysfunction following VPA exposure is important, as VPA has not previously been associated with cardiac mitochondrial dysfunction. However, VPA’s effects on hepatic mitochondria are well-established, inhibiting OXPHOS and uncoupling rat liver mitochondria through both CI- and CII-linked pathways [32], [33], [34], [35]. VPA may be expected to uncouple mitochondrial OXPHOS on account of its chemical structure. As a branched-chain saturated fatty acid, VPA could act as a protonophore that disrupts the electrochemical gradient across the inner mitochondrial membrane, decreasing the efficiency of the electron transport system [66]. It is plausible that VPA’s effect on mitochondrial coupling and OXPHOS in the liver may also occur in the heart.

VPA is known to increase the risk of developing CLZ-induced myocarditis, so exposure to both drugs was expected to cause more severe effects on OXPHOS than either drug alone [23], [24]. Combined CLZ and VPA exposure decreased O_2_ consumption during combined CI- and CII-linked as well as CII-linked OXPHOS alone (Fig. 4A). Similar to VPA alone, combined CLZ and VPA exposure caused a significant decrease in the efficiency of OXPHOS through CI- but not CII-linked respiration (Fig. 4B). The magnitude of the effect of combined drug exposure appeared to be similar to that observed following exposure to either drug alone (Fig. 2, Fig. 3, Fig. 4). Importantly, this study lacks the statistical power to make comparisons between drug effects where data is unpaired. Our observation that combined treatment does not exacerbate CLZ’s effect on respiration suggests that VPA may be acting as a substrate for β-oxidation, therefore attenuating rather than exacerbating the effect of CLZ on mitochondrial O_2_ consumption. However, VPA’s effect on mitochondrial coupling may still exacerbate the effect of CLZ on ATP production. Further investigation to clarify the effects of CLZ and VPA on ATP production and mitochondrial membrane potential would be essential in clarifying this seemingly contradictory observation.

This study focused on the acute effects of CLZ and VPA at supratherapeutic concentrations. The extent to which OXPHOS was impaired was severe, decreasing O_2_ consumption to a similar extent as what has been observed in an animal model of cardiomyopathy prior to overt heart failure [67]. To our knowledge, the time-dependent effect of CLZ and VPA on mitochondrial function have not been investigated. It would be important to consider the effect of both CLZ and VPA on human cardiac mitochondria over a longer period of exposure and at concentrations closer to the therapeutic target ranges in future studies, to determine the role of mitochondrial dysfunction in the development of cardiotoxicities. Considerable work has already gone into the development of international guidelines regarding the safer use of CLZ, considering the effects of age and ancestry on drug metabolism to develop tailored titration protocols for particular patient groups [68], [69], [70]. We believe that mitochondrial function should also be considered in this context, both as an indicator of potential risk prior to starting treatment and as a therapeutic target should cardiotoxicity occur.

In conclusion, our study provides evidence that both CLZ and VPA, alone and in combination, produce mitochondrial dysfunction in isolated human cardiac tissue, contributing to the development of cardiotoxicities [62]. Our study is the first to examine the effects of combined CLZ and VPA exposure on mitochondrial function and highlights the need to understand further the functional consequences of these drugs in the heart, as well as identify their specific mitochondrial targets, to inform the safer use of these drugs.

## CRediT authorship contribution statement

**Burns Kathryn E.:** Writing – review & editing, Supervision, Conceptualization. **Groenewald Amanda:** Writing – review & editing, Writing – original draft, Visualization, Investigation, Formal analysis. **Ward Marie-Louise:** Writing – review & editing, Supervision, Conceptualization. **Power Amelia S.:** Writing – review & editing, Supervision, Formal analysis, Conceptualization. **Tingle Malcolm D.:** Writing – review & editing, Supervision, Conceptualization.

## Declaration of Competing Interest

The authors declare that they have no known competing financial interests or personal relationships that could have appeared to influence the work reported in this paper.

## Data Availability

Data will be made available on request.
