# Supplementary material for: Acute exposure to clozapine and sodium valproate impairs oxidative phosphorylation in human cardiac mitochondria
Source: Toxicol Rep. 2025 Mar 5;14:101990. doi: 10.1016/j.toxrep.2025.101990 (PMC11946755; doi:10.1016/j.toxrep.2025.101990)
Supplement: Supplementary file 1 — Supplementary material [file mmc1.docx]

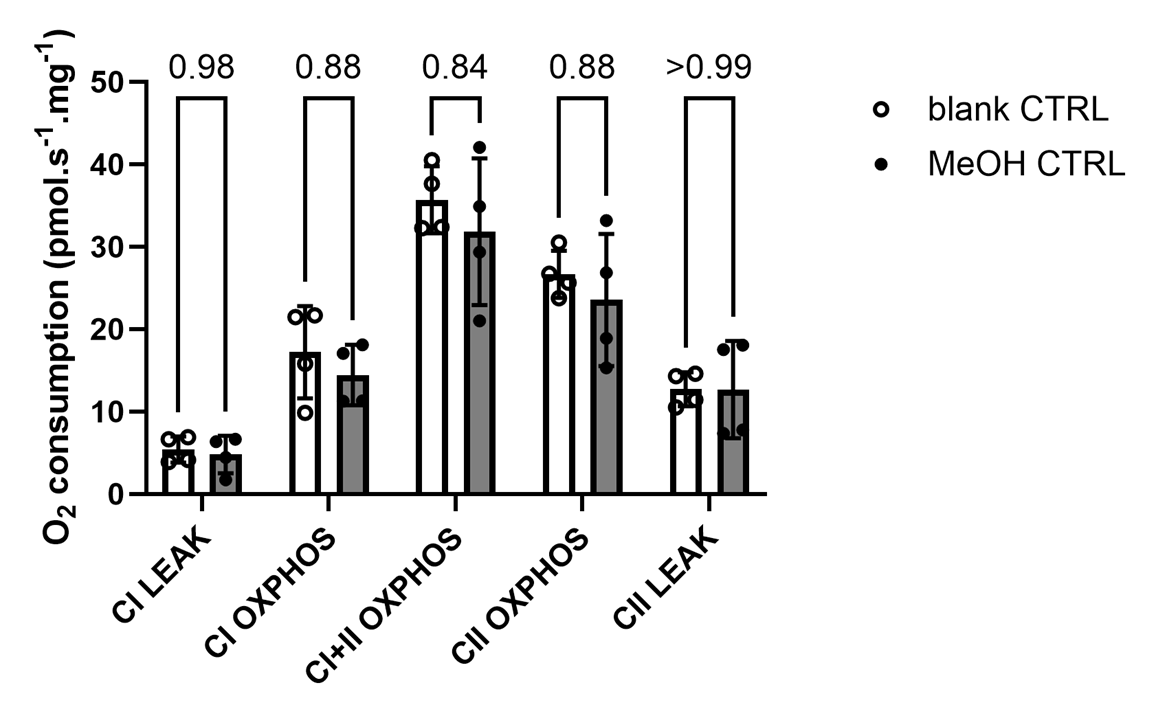


**Supplementary Fig. 1.** Mitochondrial respiration in permeabilised human atrial fibres unaffected by methanol used for drug delivery.

No differences in mitochondrial respiration were observed following a 30-minute incubation with a blank (MiR05) or methanol (MeOH, 1 % v/v) control solution (CTRL). CI- and/or CII-linked LEAK or OXPHOS were induced by the sequential titration of substrates and inhibitors after the incubation period before measuring O_2_ consumption. Residual O_2_ consumption following the addition of antimycin A was subtracted from each measure. Data are presented as mean ± SD (n = 4). P values are reported from a repeated measures two-way ANOVA with Holm-Sidak’s multiple comparisons tests.
